# Editorial: Clearance Pathways for Amyloid-β. Significance for Alzheimer's Disease and Its Therapy

**DOI:** 10.3389/fnagi.2017.00339

**Published:** 2017-10-27

**Authors:** Roxana O. Carare

**Affiliations:** Faculty of Medicine, University of Southampton, Southampton, United Kingdom

**Keywords:** clearance, amyloid, neprilysin, perivascular pathways, microglia, LRP1

Alzheimer's disease affects 9.5 million people in Europe (Alzheimer Europe) and this figure is projected to rise. Since the risk of developing late onset sporadic Alzheimer's disease is higher in patients with diabetes or hyperlipidemia in mid-life and there are clear associations between lipid-regulating genes and Alzheimer's disease, Sato and Morishita discuss the correlations between lipid, glucose and energy related pathways and the hallmarks of neurodegeneration Further, Gupta and Iadecola discuss the correlations between atherosclerosis, arteriosclerosis and the development of Alzheimer's disease. Accumulation of Aβ and tau protein in the brain in Alzheimer's disease suggests that there is an age and disease-related failure of elimination of Aβ and tau from the brain, as reviewed by Gallina et al. in this special topic. In this topic, Thal reviews the evidence for the biochemical properties of Aβ related to its potential for elimination. He concludes that even small amounts of Aβ oligomer intermediates and fibrils as well as posttranslational modifications of Aβ are poorly eliminated from the brain.

It is known that Aβ is removed from the brain by various mechanisms including degradation by neprilysin (NEP) and other enzymes, cellular uptake and by absorption into the blood via low-density lipoprotein receptor-related protein 1 (LRP1) as discussed by Ramanathan et al. The role of insulin in the elimination of Aβ is discussed in the context of evidence from epidemiological studies and molecular biology demonstrating the different interactions that occur between insulin and transendothelial clearance of Aβ (Vandal et al.). In familial Alzheimer's disease, different mutations can increase the production of mutant forms of Aβ or increase the ratio of the more fibrillogenic Aβ42 relative to Aβ38-40, as discussed by Li et al.

In this topic, Grimm et al., discusses the subcellular location and activity of the Zinc metalloprotease neprilysin. Neprilysin is expressed in neuronal axons and synapses, as well as activated glial cells and is able to degrade monomeric Aβ, but its role in degrading oligomeric forms of Aβ is still under debate. There are reduced levels of NEP in human aged and Alzheimer brains (Miners et al., 2008). The Amyloid Precursor Protein intracellular domain (AICD) and its involvement in the regulation of NEP with a wider look at other genes regulated are discussed. The role of astrocytes and microglia in eliminating Aβ from the brain is discussed in this topic by Ries and Sastre. Proteases such as neprilysin (NEP), endothelin-converting enzyme (ECE), insulin degrading enzyme (IDE), matrix metalloproteases (MMPs), cathepsin B (CAT-B) as well as chaperones apolipoprotein E (ApoE), apolipoprotein J (ApoJ), α-2 macroglobulin (α2-M), α1-antichymotrypsin (ACT) are all discussed. The mini review also covers receptors located in the surface of glial cells such as lipoprotein receptor-related protein 1 (LRP-1), scavenger receptors (SR), formyl peptide receptors (FPR), macrophage receptor with collagenous structure (MARCO), receptor for advanced glycation end products (RAGE) and triggering receptor expressed on myeloid cells 2 (TREM-2). Zhao et al. discuss emerging concepts on the up-regulation of an inducible microRNA-34a in both Alzheimer's and Parkinson's diseases that drive the down-regulation of the amyloid sensing- and clearance receptor protein TREM2.

Aβ is also eliminated from the brain along intramural periarterial drainage (IPAD) pathways in the walls of cerebral arteries (Hawkes et al., 2014). In this topic, Morris et al. demonstrate that the basement membranes of capillaries and the basement membranes surrounding smooth muscle cells of arteries form the IPAD pathways of interstitial fluid and Aβ from the brain. The IPAD pathways are small and inaccessible resulting in difficulty for their examination in experimental neuroanatomy. Computational modeling is a method that can accurately simulate biological processes and in this topic, Alexandra Diem et al demonstrates that computational modeling of IPAD is possible and IPAD along basement membranes is a process that is faster compared to diffusion in the extracellular spaces (Diem et al.).

One challenge in experimental neuropathology is translation. In this topic, Humpel presents evidence for the research applications of organoytpic brain sections of 120 μm thickness from 9 month old adult wildtype and transgenic mice expressing amyloid precursor protein (APP) harboring the Swedish K670N/M671L, Dutch E693Q, and Iowa D694N mutations; APP_SDI and cultured for 2 weeks.

The route by which Aβ from the brain parenchyma reaches the CSF in the subarachnoid space is unclear as is the selectivity of this route for different molecular species of Aβ or tau. In this special topic, McIntee et al. from the Jorge Ghiso lab demonstrate that monomeric and low molecular weight oligomeric [^125^I]Aβ1–40 injected into the brain parenchyma are rapidly removed at 5, 30, and 60 min post-injection. Degradation of Aβ in the parenchyma occurred within 5 min and approximately 3% of intra-cerebrally injected ^125^I-Aβ was found in CSF after 5 min, with 13% 1 h post-injection. This work highlights the need for further assessment of the barrier properties at the interface between the meninges and the cerebral parenchyma as regulators of the CSF protein biomarkers reflecting brain pathology.

The topic addresses the new therapeutic attempts and challenges for Alzheimer's disease, summarized by the groups of (Morrone et al.). Saito and Ihara present experimental evidence that cilostazol, a selective inhibitor of type 3 phosphodiesterase improves intramural periarterial drainage of Aβ. Since the publication of the topic, cilostazol has entered phase II clinical trials. Several immunization trials and approaches to reduce the synthesis of Aβ using β-site APP Cleaving Enzyme (BACE) inhibitors are taking place now and most of them rely on efficient elimination of the excess fluid and solubilised Aβ from the aged Alzheimer brains.

## Author contributions

The author confirms being the sole contributor of this work and approved it for publication.

### Conflict of interest statement

The author declares that the research was conducted in the absence of any commercial or financial relationships that could be construed as a potential conflict of interest.
